# Evaluation of Laser Speckle Contrast Imaging for the Assessment of Oral Mucosal Blood Flow following Periodontal Plastic Surgery: An Exploratory Study

**DOI:** 10.1155/2017/4042902

**Published:** 2017-01-23

**Authors:** Eszter Molnár, Bálint Molnár, Zsolt Lohinai, Zsuzsanna Tóth, Zoltán Benyó, Laszló Hricisák, Péter Windisch, János Vág

**Affiliations:** ^1^Department of Conservative Dentistry, Faculty of Dentistry, Semmelweis University, Szentkirályi Utca 47, Budapest 1088, Hungary; ^2^Department of Periodontology, Faculty of Dentistry, Semmelweis University, Szentkirályi Utca 47, Budapest 1088, Hungary; ^3^Institute of Human Physiology and Clinical Experimental Research, Faculty of Medicine, Semmelweis University, Tűzoltó Utca 37-47, Budapest 1094, Hungary

## Abstract

The laser speckle contrast imaging (LSCI) is proved to be a reliable tool in flap monitoring in general surgery; however, it has not been evaluated in oral surgery yet. We applied the LSCI to compare the effect of a xenogeneic collagen matrix (Geistlich Mucograft®) to connective tissue grafts (CTG) on the microcirculation of the modified coronally advanced tunnel technique (MCAT) for gingival recession coverage. Gingival microcirculation and wound fluid were measured before and after surgery for six months at twenty-seven treated teeth. In males, the flap microcirculation was restored within 3 days for both grafts followed by a hyperemic response. During the first 8 days the blood flow was higher at xenogeneic graft comparing to the CTG. In females, the ischemic period lasted for 7–12 days depending on the graft and no hyperemic response was observed. Females had more intense and prolonged wound fluid production. The LSCI method is suitable to capture the microcirculatory effect of the surgical intervention in human oral mucosa. The application of xenogeneic collagen matrices as a CTG substitute does not seem to restrain the recovery of graft bed circulation. Gender may have an effect on postoperative circulation and inflammation.

## 1. Introduction

In today's periodontal plastic surgery numerous flap designs with various grafting alternatives (autograft, allograft, or xenogeneic materials) are routinely applied. Graft exposure during soft and hard tissue augmentation might occur before there would be any chance for graft vascularization to take place due to wound healing disturbances and a lack of primary intention healing. Compromised flap circulation could result in flap failure, which can be avoided by a proper flap design [1, 2] and tension-free flap advancement [3]. In order to minimize trauma to the surrounding tissues (i.e., the flap) it is recommended to use the least invasive method for flap preparation, which may also protect the underlining graft tissue and support quick vascularization. These considerations led to the development of a minimally invasive flap design for root coverage surgery, namely, the tunnel technique [4, 5].

The application of a connective tissue graft (CTG) in combination with a coronally advanced flap (CAF) delivers the most predictable outcomes in the treatment of gingival recessions [6]. However, the application of autologous tissues inevitably requires harvesting from a donor site, most commonly from the palate with the single incision technique. This increases the duration of the surgery and patient morbidity and requires extended postoperative medication [7]. Moreover, the accessible pool of available tissues in a single harvesting procedure is limited and the regeneration of the palatal tissues for repeated harvesting takes several months [8]. Xenogeneic materials combined with CAF also represent a viable alternative for treating gingival recessions [9, 10] with less complaints [11]. Geistlich Mucograft (Geistlich Pharma AG, Wolhusen, Switzerland) is a porcine collagen matrix recommended as an alternative to connective tissue grafting for root coverage surgery in combination with CAF or modified CAF (MCAT) [9, 12, 13]. Geistlich Mucograft was reported to have delayed revascularization along with a prolonged vascularization of the surrounding tissues (i.e., the wound bed) [12]. Generally, prior to complete revascularization, the nutritive supply of the graft occurs by diffusion from the surrounding tissues [14–16]. Therefore, blood flow in tissues in the close vicinity of the graft may be even more important in successful wound healing.

Recently, a new noninvasive two-dimensional method, namely, laser speckle contrast imaging (LSCI), has been introduced to evaluate the microcirculation of tissues [17]. Clinical studies are suggesting that this technique may be a useful tool for assessing proper circulation during surgical intervention [18–20] and evaluating wound healing [21, 22], but it has not been tested in human oral mucosa yet.

Our primary aim was to apply LSCI to characterize the kinetics of blood flow changes of MCAT and determine the optimal setting of the wound healing monitoring in human subjects. Our secondary aim was to test whether application of xenogeneic (Geistlich Mucograft) material may delay recovery of microcirculation of the MCAT flap comparing to the gold standard autograft (CTG).

## 2. Materials and Methods

### 2.1. Participants

Eight subjects (four women and four men) exhibiting multiple Miller Classes I and II gingival recessions (Multiple Adjacent Recession Type Defects, MARTD) were recruited. All subjects had a thin gingival biotype assessed by thickness which had been measured using Kerr file preoperatively. They were in good general health, and their mean age was 35 (age ranged between 26 and 46). Exclusion criteria were pregnancy, smoking, general diseases; furthermore, the subjects were not allowed to take any antibiotics, anti-inflammatory drugs, systemic steroids, bisphosphonates and any other medicine possibly influencing mucosal wound healing, or any other products in the preceding three months. The patients had good oral hygiene; PSRs (Periodontal Screening and Recording) were zero at each sextan, and full mouth plaque and bleeding scores were maintained below 20% throughout the study. Each subject received written information about the surgery and the subsequent measurements, enabling them to give a written informed consent. The study was carried out in accordance with the Declaration of Helsinki. Ethical approval was granted on October 29, 2014, by the Hungarian authority called Committee of the Health Registration and Training Center (approval number: 034310/2014/OTIG). The study was registered in the ClinicalTrials.gov (Identifier: NCT02540590).

### 2.2. Surgery

MARTDs were treated with MCAT (reported elsewhere: [23]) by an experienced periodontist. Two types of grafts were used during the surgeries: either a subepithelial connective tissue graft (CTG) removed from the palate or a xenogeneic collagen matrix (Geistlich Mucograft). Five patients received both grafts in a split-mouth design. Three patients were treated only at one surgical site (two of them received Geistlich Mucograft and one received CTG). Immediately before surgery, root scaling was performed with hand instruments, and a flow composite was applied coronally at the contact points for later suture suspension. Patients were instructed to follow postoperative regimes. In the control period, patients had to rinse with mouthwash containing 0.2% chlorhexidine (Curasept 220, Curaden, Switzerland) until 14 days after the surgery. Manual brushing at the treated sites was prohibited until suture removal. Supragingival debridement was performed at operation sites using a scaler and chlorhexidine-soaked cotton balls. Patients were given systemic antibiotics postoperatively for seven days.

### 2.3. Data Collection

Clinical data collection was carried out at baseline (bsl.) and six months postoperatively. Photo documentation was prepared at all visits. Blood flow, blood pressure, and wound fluid measurements were done before the operation (baseline) and postoperatively on the following days: 1, 2, 3, 4, 5, 6, 7, 8, 10, 12, 14, 17, 30, 60, 90, 120, 150, and 180.

### 2.4. Clinical Parameters

The following clinical parameters were recorded by means of a periodontal probe at baseline and after six months: gingival recession depth (GRD0 and GRD6), gingival recession width (GRW0 and GRW6), and the width of the keratinized tissue (KT0 and KT6). The change of these parameters was calculated as follows: recession depth reduction (REC), recession width reduction (RW), and increase of the keratinized tissue in width (KT).

### 2.5. Blood Pressure Measurement

Systolic and diastolic blood pressure and pulse rate were measured with an automatic blood pressure monitor (Omron M4, Omron Healthcare Inc., Kyoto, Japan) before and after the LSCI measurements. Mean Arterial Pressure (MAP) was calculated from these values.

### 2.6. Blood Flow Measurement

Blood flow was measured at the gingiva of 52 teeth in total: at 27 sites of operated teeth (test sites) and at 25 control teeth (reference sites, 13 in female and 12 in male). Of the measured test sites, 14 were Geistlich Mucograft-treated (7 in female and 7 in male) and 13 were CTG-treated (6 in female and 7 in male).

Subjects were forbidden to brush their teeth, gargle and rinse, or eat or drink anything for 60 minutes prior to the measurements. Each patient was placed comfortably in supine position in a dental chair and was left undisturbed for a minimum of 15 minutes before any measurements were taken. The lips were retracted carefully and without tension with dental mirrors. Care was taken to ensure that the mucosal surface adjacent to the site of recording remained unstrained. All measurements were obtained at 26°C room temperature and always between 7 and 10 o'clock in the morning.

Blood flow was measured by LSCI (785 nm PeriCam PSI HR System, Perimed AB, Stockholm, Sweden). The resolution was set to 60 *μ*m/pixel. The distance from the measured surface to the LSCI instrument's objective was set to 10 cm. The LSCI instrument was connected to a computer and the measured values were displayed and recorded with a software application (PimSoft, Perimed AB, Stockholm, Sweden). The instrument was set to take snapshots of each area. Each snapshot was constructed by averaging 20 images in 2 secs in order to average out pulsatile variation in the blood flow.

**Figure 1:**
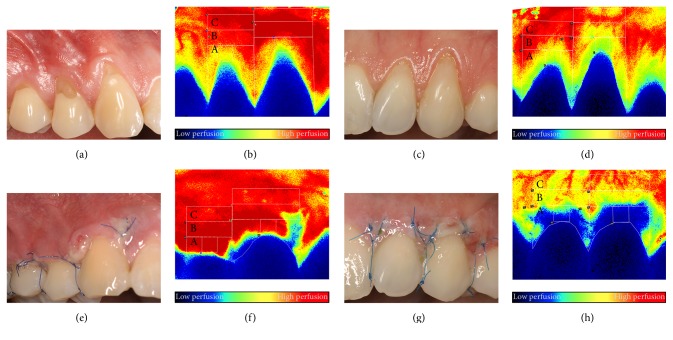
Representative photographs and LSCI images of a male (a, b, e, f) and a female (c, d, g, h) subjects. Combination of the modified coronally advanced tunnel and Geistlich Mucograft in both cases. (a, b, c, d) Images representing the preoperative perfusion. (e, f, g, h) Images showing the wound healing and perfusion 3 days postoperatively. Capital letters (A, B, and C) indicate the regions of interest for the blood flow evaluation.

Three regions of interest (ROI) were defined at each tooth as shown in Figure 1 (zone A, zone B, and zone C, moving away from the crown). The selection of regions and further steps of the data process were accomplished by blind analysis. The blood flow value of ROI was defined as the average of all the pixel perfusion values in the ROI. The approximate pixel number was 7000 for zone A and 3500 for zones B and C. According to the point density it spanned 20 mm^2^ and 14.5 mm^2^, respectively. Blood flow was expressed in an arbitrary value called Laser Speckle Perfusion Unit (LSPU).

### 2.7. Wound Fluid Measurement

After the blood flow measurements, the relative volume of the wound fluid (WF) was assessed by Periotron 8000 (OraFlow Inc., NY, USA) with a filter paper (Periopaper, OraFlow Inc., NY, USA). The surface of the teeth was gently dried and Periopaper was placed for 10 seconds close to the orifice of the sulcus in the midbuccal area of treated and nontreated teeth. The values are shown in Periotron Scores (PS).

### 2.8. Statistical Analysis

Data in the text and the figures are presented as mean ± standard error of mean (SE). The blood flow changes were analyzed by a mixed-model approach. For pairwise comparison the* p* values were adjusted by the Benjamini and Hochberg method in order to control the false discovery rate in multiple testing. To determine the association between the clinical outcome and baseline clinical parameters and between the blood flow and WF data, Spearman's correlation coefficients were calculated. The differences in clinical parameters between grafts and between genders were tested using the nonparametric Mann–Whitney* U* test. Statistical evaluation was carried out by IBM SPSS Statistics for Windows (Armonk, NY: IBM Corp., USA).

## 3. Results

### 3.1. Systemic Blood Pressure and Blood Flow at the Reference Sites

No significant change was found in MAP throughout the observation period (90 ± 2.6 mm Hg at bsl. versus 85 ± 2.9 mm Hg on day 180). There was no correlation between MAP and blood flow in either zone (zone A: *r* = −0.115, *p* = 0.140; zone B: *r* = −0.130, *p* = 0.096; zone C: *r* = −0.057, *p* = 0.471). Therefore, the blood flow values were used instead of vascular resistance in the following analysis.

### 3.2. Blood Flow at the Treated Sites on the Days following the Surgery in Zone A

**Figure 2:**
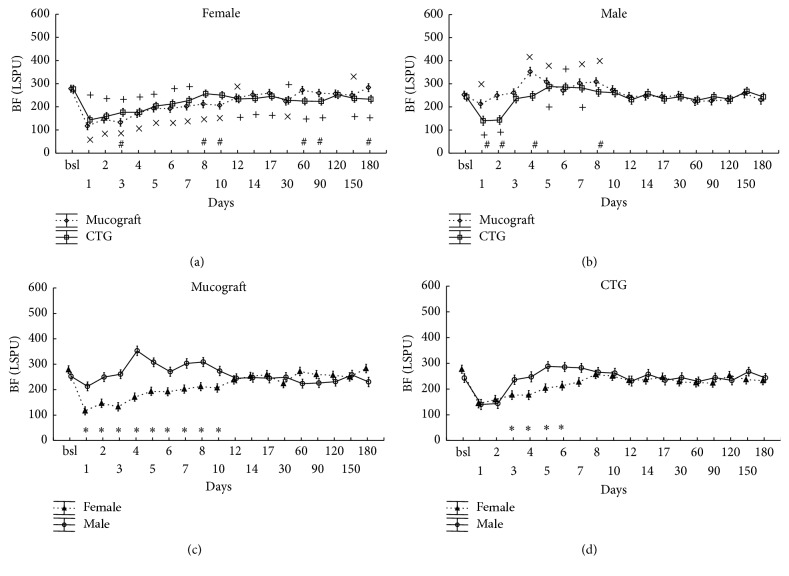
Time-course of the changes of gingival blood flow (BF) in zone A, expressed in Laser Speckle Perfusion Unit (LSPU). Time points include preoperative data (bsl.) and postoperative days (1 to 180). Data are presented as means ± SE. In (a, b), statistically significant differences in the postoperative values versus bsl. are indicated by × in Geistlich Mucograft (*n* = 14) and by + in CTG (*n* = 13). The differences between grafts at the respective time points are indicated by #. In (c, d), the same data are shown in a different grouping as gender differences are depicted separately for Geistlich Mucograft and for CTG. *∗* indicates significantly different time points between the genders. ×, +, #, and *∗* mark significance levels of *p* < 0.05 after being adjusted by the Benjamini and Hochberg method.

The statistical analysis showed that not only the graft (graft × time: *p* < 0.001) but also gender has a strong influence (gender × time: *p* < 0.001) on the blood flow of the healing mucosa. Furthermore, a significant interaction was observed between gender, graft type, and time (*p* < 0.001). The data were therefore split into two subgroups based on gender in addition to the two graft types (Figure 2).

In females, blood flow at the treated teeth dropped significantly, approximately to half of the baseline values in the case of both Geistlich Mucograft and CTG on the first day after the surgery (Figure 2(a)). After day 2, blood flow increased towards the baseline but remained below it until day 12 in Geistlich Mucograft patients and until day 7 in CTG patients. Over the six-month period, there was only a slight difference in flap circulation between the two graft groups.

In males, contrary to females, there were marked differences in blood flow between the two grafted sites (Figure 2(b)). Blood flow at Geistlich Mucograft-treated sites returned to baseline on day 2 and a hyperemic response occurred from day 4 to day 8. At CTG-treated sites, blood flow returned to baseline on day 3, and a reduced and shorter hyperemic response developed between day 5 and day 7. Perfusion at Geistlich Mucograft-treated sites significantly exceeded the corresponding values for CTG on days 1, 2, 4, and 8 in males.

On Figures 2(c) and 2(d), the same data as on the upper panels were reconstructed for comparison between the genders at each time point, separately for each graft. The blood flow values of males significantly exceed those of females between days 1 and 10 in the case of Geistlich Mucograft and from day 3 to day 6 in the case of CTG.

### 3.3. Blood Flow at the Treated Sites on the Days following the Surgery in Zone B

**Figure 3:**
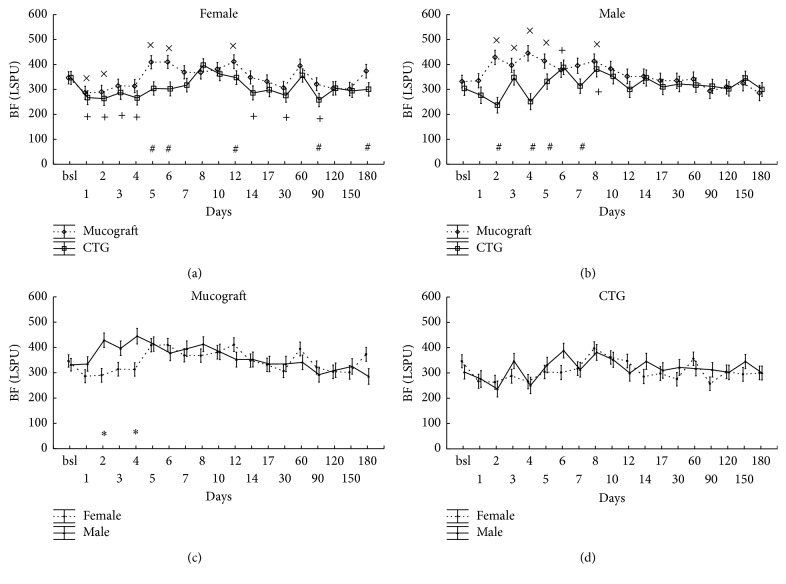
Time-course of the changes of gingival blood flow (BF) in zone B, expressed in Laser Speckle Perfusion Unit (LSPU). Time points include preoperative data (bsl.) and postoperative days (1 to 180). Data are presented as means ± SE. In (a, b), statistically significant differences of the postoperative values versus bsl. are indicated by × in Geistlich Mucograft (*n* = 14) and by + in CTG (*n* = 13). The differences between grafts at the respective time points are indicated by #. In (c, d), the same data are shown in a different grouping as gender differences are depicted separately for Geistlich Mucograft and for CTG. *∗* indicates significantly different time points between the genders. ×, +, #, and *∗* mark significance levels of *p* < 0.05 after being adjusted by the Benjamini and Hochberg method.

Similarly to zone A, the analysis was done on the level of gender × graft × time (*p* < 0.001) interaction (Figures 3(a) and 3(b)). However, contrary to zone A, the effect of the graft as a main factor was strong and significant (*p* < 0.001) while gender × graft was not due to the fact that blood flow at Geistlich Mucograft-treated sites was always above CTG values regardless of gender.

The graphs in Figures 3(c) and 3(d) demonstrate that, only in the case of Geistlich Mucograft, blood flow values were significantly higher in males than in females on days 2 and 4 while no difference was detected at CTG-treated sites.

### 3.4. Blood Flow at the Treated Sites on the Days following the Surgery in Zone C

**Figure 4:**
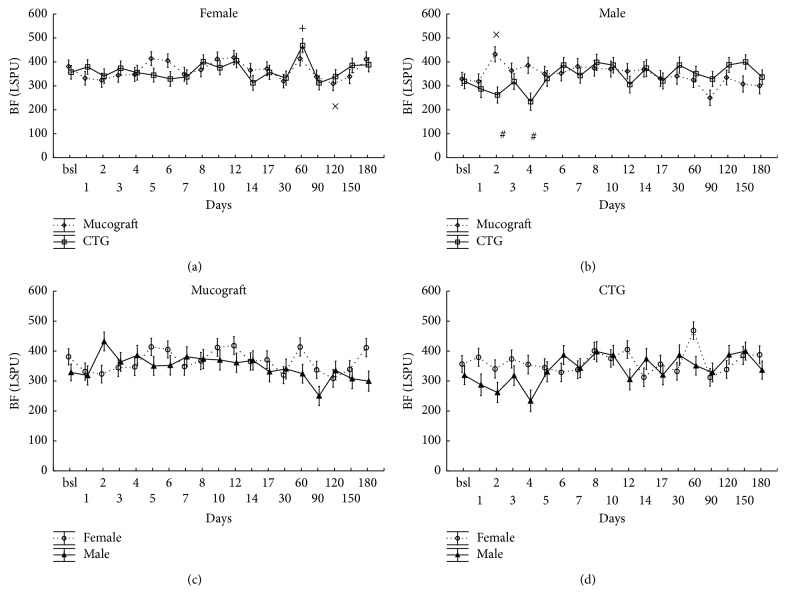
Time-course of the changes of gingival blood flow (BF) in zone C, expressed in Laser Speckle Perfusion Unit (LSPU). Time points include preoperative data (bsl.) and postoperative days (1 to 180). Data are presented as means ± SE. In (a, b), statistically significant differences of the postoperative values versus bsl. are indicated by × in Geistlich Mucograft (*n* = 14) and by + in CTG (*n* = 13). The differences between grafts at the respective time points are indicated by #. In (c, d), the same data are shown in a different grouping as gender differences are depicted separately for Geistlich Mucograft and for CTG. There were no significant differences observed between the genders. ×, +, and # indicate significance levels of *p* < 0.05 after being adjusted by the Benjamini and Hochberg method.

Blood flow in zone C was less affected by the surgery, but still the effect of time was significant (*p* < 0.001). Neither the graft nor the graft-gender interaction had an overall effect on blood flow (*p* = 0.84; *p* = 0.89). As in the case of zones A and B, the analysis showed that all interactions with the time factor (graft × time: *p* < 0.001; gender × time: *p* < 0.001; gender × graft × time: *p* < 0.001) were significant, indicating some variations in blood flow by time over the observation period (Figure 4). The graphs on the lower panels of Figure 4 demonstrate that there were no differences observed in this zone between the genders.

### 3.5. Wound Fluid Measurements

**Figure 5:**
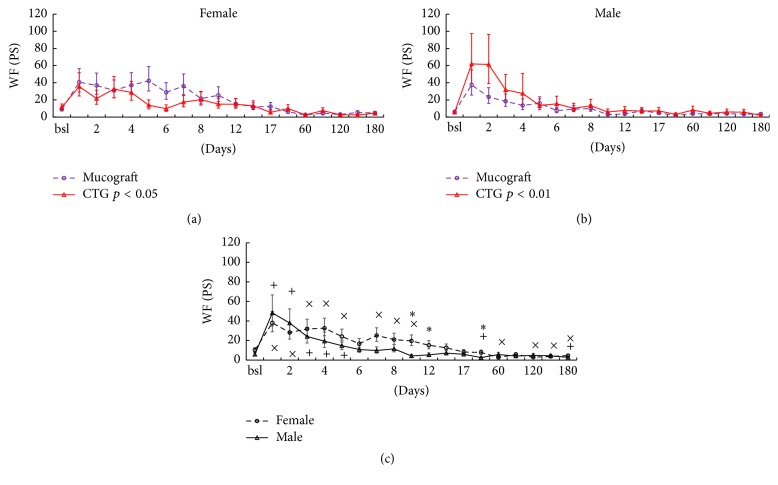
The effect of time, graft, and gender on wound fluid (WF) production. The two upper graphs (a and b) show the interaction between graft and gender in wound fluid (WF) production during the whole period, expressed in Periotron Scores (PS). The lower plot (c) shows the changes of WF production over time when the graft data were grouped. Time points include preoperative data (bsl.) and postoperative days (1 to 180). Data are presented as means ± SE. Statistically significant differences of the postoperative values versus bsl. are indicated by × in females and by + in males. The differences between the genders are indicated by *∗* (*p* < 0.05, adjusted by the Benjamini and Hochberg method).

The two main factors, graft type (*p* = 0.85) and gender (*p* = 0.13), were not significant but time (*p* < 0.001) was. Interactions between graft × time (*p* = 0.70) and graft × gender × time (*p* = 0.46) were not significant either, but the graft × gender interaction was significant (*p* < 0.001). This means that, overall, the WF production of Geistlich Mucograft-treated sites (13.8 [+2.6, −2.2] PS) exceeded that of CTG-treated sites (10.7 [+2.1, −1.8] PS) in females (Figure 5(a)). On the other hand, in males, the opposite was found: Geistlich Mucograft-treated sites had less WF (6.9 [+1.5, −1.2] PS) than CTG-treated sites (10 [+2.4, −1.9] PS) (Figure 5(b)).

As time interacted with gender (*p* < 0.001), pairwise comparisons were made at each time point (Figure 5(c)). PSs increased dramatically in both genders on the first day after surgery. They remained significantly higher than the baseline until day 10 in females and until day 5 in males. On the first two days, WF looked similar in both genders but from day 3 the values in males dropped steeper than in females. One month after the surgery, WF tended to be lower than the respective baseline values in both genders.

### 3.6. The Correlation between WF and Blood Flow

During the early healing period, blood flow in zone A showed a moderate inverse correlation with WF production on day 4 (*r* = −0.55, *p* < 0.05), day 5 (*r* = −0.49, *p* < 0.05), day 6 (*r* = −0.51, *p* < 0.05), and day 7 (*r* = −0.61, *p* < 0.01).

### 3.7. Clinical Parameters

Baseline GRD0 and GRW0 were very similar in the Geistlich Mucograft- and the CTG-treated groups (Table 1); however, the initial KT0 was significantly less in the CTG-treated group. Gains in the depth (REC) and width (RW) of the recessions were similar in the two groups. The increase in KT at Geistlich Mucograft-treated sites was significantly less than that at CTG-treated sites (Table 1).

No statistically significant differences were observed between females and males either in the baseline values or in REC, RW, and KT (data not shown).

REC and RW were positively correlated with the baseline values (*r* = 0.92, *p* < 0.001 and *r* = 0.64, *p* < 0.001). In contrast, KT was negatively correlated with KT0 (*r* = −0.79, *p* < 0.001).

## 4. Discussion

Our primary aim was to introduce the LSCI method to monitor the microcirculation of the oral mucosa after periodontal plastic surgery interventions. Imaging technique simultaneously displays several areas of the flap contrary to the single-point laser Doppler flowmetry. Another unique property of LSCI is the rapid imaging which reduces movement artefact and decreases time of each measurement session especially when multiple images within a mouth has to be captured. These features facilitate patient compliance during many visits. This new method has not been tested before on postoperative mucosal flap; therefore we had no data available about the intraday and interday variability. In order to get the best estimation of the time-course we performed multiply repeats on each day and we made measurements on numerous days during the wound healing period. Importantly due to the great number of measurements approximately 8000 ROIs (~1000 per patient, ~150 per tooth-site) were drawn manually and resulted in hard work of data processing. In this exploratory study we managed to define the most characteristic days (1, 3, 7, and 10) for the flap circulation which could decrease the necessary session of the measurements in further high scale studies. The split-mouth design is also a good way to decrease the number of the patients involved as it decreases the error rate due to the low relative standard deviation (<7%) of the gingival sites within a patient. This data also suggests that the effect of surgery intervention on the variability between parallel tooth sites within a surgical area managed to be kept fairly standardized probably due to the single experienced operator. Involving some reference sites to normalize the values at the test sites has only slightly decreased the error rate; thus it could be dropped. Contrary to the LSCI method the single-point laser Doppler technique suffers from the difficulty in repositioning of the probe during day by day follow-up and it is very sensitive to the measurement distance and angulation [21, 24–26]. In our study the spatial variability was decreased by using thousands of pixels for each ROI spanning 10–20 mm^2^ and the instrument was set to fixed focal distance. Overall these carefully settings in our design resulted in low variability among patients (<11%), which promoted the better power for evaluation of the between-group effect such as the gender. The LSCI method was able to capture not just the massive effect of surgical intervention but also small differences in the surgical technique used. We assume that this method may help to understand physiological and pathophysiological changes during mucosal healing. Understanding the mechanism would help us to readjust postoperative care and select the best available surgical techniques, such as incision and flap design, suture, and graft size and type.

According to our results, the most apical area (zone C) was the least influenced by the surgery, as this was repositioned directly to vital tissues without an intermediate graft. Moreover, it is a more distensible mucosal area with the best collateral circulation. On the other hand, the most severe ischemia was observed in the marginal area (zone A) due to the intrasulcular incisions which cut off the main collateral circulation (with the periodontal plexus) of this area. Furthermore, the suspended sutures and the underlying grafts cause probably the highest tension in this area. Blood flow in this area returned to the baseline level within 14 days in all cases and in some cases much earlier. In addition, in males, a hyperemic response was also observed after the hypoperfusion between day 4 and day 8 postoperatively.

Only a few studies investigated the blood flow of the flap in the oral mucosa. A study of four dogs [27] showed that the simple elevation of mucoperiosteal flaps causes a postoperative ischemia for seven days before blood flow would return to baseline. In a human study [28] using laser Doppler flowmetry, a postoperative hyperemic response was observed one day after the periodontal flap surgery. In this study the vascular response represented the mean of the two sex groups and neither of these studies applied graft material for the surgery. Histological observations suggested that tissue revascularization begins in mucosal flaps as early as two or three days postoperatively [29, 30]. Vascularity regained its normal level in 10 days after surgery if there was no significant interface (i.e., any grafting material) between the bone and the repositioned flap [31, 32]. However, if bone grafts and membranes separated the flap from the bone it took three weeks for vascularity to be normalized [33], which highlights the role of reuniting the alveolar and periodontal plexus to the mucosal one.

Furthermore, xenogeneic matrices were not bearing a vasculature contrary to autologous grafts, where revascularization can occur earlier by inosculation [16, 34]. The vascularization of the xenogeneic graft area only begins after the graft is almost disrupted which takes months [35, 36]. In animal study [12] the vascularization begins very slowly and sparsely after application of Geistlich Mucograft collagen matrix. The quick restoration of the blood flow long before the expected revascularization confirmed that MCAT only minimally compromised the mucosal vascular architecture and the full graft vascularization is not necessary for mucosal regeneration. The early recovery of flap circulation helps to cover and protect the grafted area and to promote tissue integration. Similarly, it was observed previously [37] that a careful and less invasive surgical approach—for example, employing microsurgical rather than macrosurgical techniques—may better maintain circulation and speeds up revascularization. This also resulted in better clinical performance [5, 38]. Favorable flap circulation and the relatively intact periosteal plexus could provide a good double-layered recipient bed for the grafts. This serves as a good nutritive supply for both autogeneic and xenogeneic grafts by imbibition until new vessels develop within the grafts.

In spite of the fact that flap circulation slightly favored the xenogeneic matrix, the CTG resulted in similar root coverage compared to Geistlich Mucograft, with comparable mean baseline recession depth in both groups. This is in accordance with the findings of randomized clinical trials (RCT) [13, 39] where the percentage of root coverage by Geistlich Mucograft remained only slightly below that of the CAF + CTG. Although the gain in keratinized tissue width was slightly less in the Geistlich Mucograft than in the CTG group, which is also confirmed by an RCT [13], another RCT found no differences [10, 39]. Mean KT at baseline was slightly different across the different groups which may have some effect on the clinical outcome.

Interestingly, unlike the moderate effects of the graft types, gender made a considerable impact on flap circulation. This was an unexpected result compelling us to split our data into two subgroups. Ignoring the gender factor would have resulted in a failure to correctly assess the recovery time of blood flow as the two curves would have cancelled each other out. Furthermore, the effect of the different grafts on microcirculation in both zones A and B would not have been assessed either. Males had an ischemic phase lasting a few days, followed by a hyperemic response in the marginal gingival zone whereas females were characterized by a slower recovery of the blood flow. All baseline clinical parameters of the gingiva including crevicular fluid and tissue morphology (e.g., recession depth, recession width, width of the keratinized mucosa, and thickness of the keratinized mucosa) were similar in the two gender groups. These suggest that neither the initial subclinical inflammation nor the morphology of the gingival recession can explain the gender-specific postoperative alteration in blood flow. The differences in blood flow between the genders were less pronounced in zone B and disappeared in zone C, implying that the gender effect is more important in the marginal zone where the circulation is most severed. To the best of our knowledge, there are no data available on the effect of gender on the flap microcirculation on the oral mucosa. Clinical observations [40–43] and findings in an experimental excisional palatal wound model [44] suggest that mucosal wound healing is faster in males than in females; however blood flow was not measured in these studies. It can only be supposed that better blood flow recovery may have facilitated wound healing in males.

The MCAT recovered earlier than the expected complete revascularization period suggesting that higher blood flow in males occurred due to the vasodilation of the remaining vessels. We can further suppose that there may be differences in terms of vascular reactivity between the genders. After surgery blood flow was reduced as the vascular supply of the flap was compromised which may result in low-flow mediated vasoconstriction. In the brachial artery an in vivo prolonged low-flow condition augments vasoconstriction during occlusion and attenuates hyperemic response [45] and low-flow mediated vasoconstriction seems to be more intense in women [46].

Arteriogenesis or the so called “collateralization” is another important mechanism to maintain perfusion in case of reduced vascularity before neovascularization can be completed. This involves a proliferative increase in the diameter and length of the arterioles to compensate for the reduced perfusion of the flap [47]. In an ischemic limb mouse model blood flow recovered faster in males and more alpha smooth muscle cell positive vessels—arterioles—could be found [48] suggesting greater arteriogenesis. Furthermore, higher maximal vasodilation in response to acetylcholine and nitroglycerin in male ischemic limbs was also recorded. Similarly, in patients with stable angina and chronic total occlusion of at least one major epicardial coronary artery [49] and in a rat myocardial infraction model the remodeling of arteriolar vessels (arteriogenesis) was found to be reduced in females [50]. There is evidence that the gingiva reacts by collateralization to pathophysiological stimuli such as periodontitis [51–53]. As a conclusion we hypothesized that in the gingival tissues of males there may be more native collaterals and/or increased collateralization after surgery and/or higher reactivity to vasodilation agents which might be attributable to gender differences.

Until the recovery of graft vascularization, a vascular leak maintains the nutritive supply of the graft via imbibition [14, 16]. However, not only the graft but also the distal part of the mucosal flap is supplied by nutrition via extravasation in the early ischemic period of healing [27]. In the present study, wound fluid production was measured in order to indirectly and noninvasively assess vascular permeability. Interestingly, blood flow recovery showed a correlation with recovery to normal tissue transudation. In males, this happened earlier—within 2-3 days for blood flow and within six days for wound fluid—whereas in females, it was 8–14 days and 12 days, respectively. As in the case of blood flow in the flap (in zone A), differences in vascular leakage between the grafts showed some gender specificity. In females, Geistlich Mucograft-treated sites had higher fluid production which may be a compensatory mechanism of the lower blood flow while in males the tissue may require less diffusive nutrition due to the superior blood flow compared to CTG-treated sites. Similarly, it was observed in male mice [15] that skin graft angiogenesis peaked at 10 days after grafting and this was coincident with maximum vascular leakage.

## 5. Conclusions

The LSCI method seems to be feasible way to characterize the postoperative flap circulation in oral mucosa. It can be concluded that the application of both grafts resulted in excellent recirculation patterns in the flap. Gender was the most substantial influencing factor. Males showed a more rapid reestablishment of mucosal blood flow. Further study is necessary to investigate the mechanism of the gender-specific blood flow regulation in the healing mucosal flap. We found that earlier blood flow recovery is strongly associated with earlier normalization of vascular permeability. It is conceivable that the opposite changes observed in blood flow versus vascular permeability are equally able to ensure the supply required for the tissues to heal. This, however, did not influence the apparent clinical outcome; favorable recession coverage could be achieved with both CTG and Geistlich Mucograft in males and females.

## Figures and Tables

**Table 1:** Gingival recession characteristics obtained at baseline and six months after the surgery.

	Geistlich Mucograft (*n* = 14 sites)	CTG (*n* = 13 sites)
	mean ± SE in mm	mean ± SE in mm
GRD0	2.4 ± 0.23	2.8 ± 0.27
GRW0	3.2 ± 0.28	3.2 ± 0.26
KT0	3 ± 0.41^#^	1.4 ± 0.35
REC	1.9 ± 0.29	2.6 ± 0.27
RW	2.1 ± 0.47	2.7 ± 0.40
KT	−0.7 ± 0.29^#^	0.7 ± 0.41

GRD0, gingival recession depth at baseline; GRW0, gingival recession width at baseline; KT0, width of the keratinized tissue at baseline; REC, recession depth reduction; RW, recession width reduction; KT, increase in the width of the keratinized tissue. # represents the statistically significant differences between graft types; *p* < 0.05.
